# *Stenotrophomonas maltophilia* Meningitis in a Term Healthy Neonate: A Case Report and Literature Review

**DOI:** 10.1155/2018/1543934

**Published:** 2018-06-13

**Authors:** Judy Ibrahim, Nadia Hamwi, Hala Rabei, Mohamed Abdelghafar, Zahraa Al-Dulaimi, Hossam Al Tatari

**Affiliations:** ^1^Department of Academic Affairs, Tawam Hospital, Al Ain, UAE; ^2^Dubai Medical College, Dubai, UAE; ^3^Pediatrics Infectious Diseases Department, Tawam Hospital, Al Ain, UAE

## Abstract

*Stenotrophomonas maltophilia* is an environmental bacterium of growing concern due to its multidrug resistance and pathogenic potential. It is considered an opportunistic pathogen of nosocomial origin most of the time, targeting a specific patients' population. We describe a case of a previously healthy full-term neonate who was found to have *S. maltophilia* meningitis and was successfully treated with a combination of Trimethoprim-Sulfamethoxazole and Ciprofloxacin.

## 1. Introduction

*S. maltophilia* was first described by Hugh and Ryschenkow in 1961 [1] as a Gram-negative, glucose nonfermentative aerobic rod bacteria. It was previously known as *Pseudomonas maltophilia* and *Xanthomonas maltophilia*. *S. maltophilia* is known as a low-virulence commensal organism that was typically isolated within hospitals and health-care facilities. Therefore, it is usually believed to be an opportunistic nosocomial pathogen. However, community-acquired *S. maltophilia* infections have been reported. *S. maltophilia* is now of rising importance since it is a multidrug resistant organism that is associated with high morbidity and mortality. It is known to cause a wide spectrum of serious infections, including bacteremia, endocarditis, ocular infections, urinary tract infections, skin and soft tissue infections, pyomyositis, sepsis, and meningitis [2]. *S. maltophilia* meningitis in pediatrics is very rare with only very few cases reported since 1977.

## 2. Case Presentation

This was a 13 days old baby boy, who was born via spontaneous vaginal delivery at term in our tertiary care hospital without any postnatal complications. He was discharged 24 hours after delivery. He was brought back to our ER with left eye purulent discharge, which was noticed since birth, and swelling of his left upper eyelid of 2 days duration.

There was no associated fever or history of decreased level of activity or feeding. There was no history of rashes or seizures.

The pregnancy course was remarkable only for gestational diabetes and the fact that the mother had a history of vaginal discharges, which was treated as vaginal candidiasis during the last trimester. *Group B streprococcus* screening on the 37th week of gestation was negative. Similarly, HIV and hepatitis B serology were negative one day prior to delivery. There was no maternal history of genital lesions, vesicles, or ulcers.

Examination was normal apart from the purulent eye discharge & swelling of the left eye upper eyelid. The eye secretions were yellowish sticky, copious, and profound. Fontanelles were soft & primitive reflexes were present and normal.

Due to suspicion of gonococcal ophthalmia neonatorum, a full septic workup was obtained including CBC, blood culture, urine analysis and culture, CSF analysis and culture, and left eye swab for culture and *Chlamydia* antigen (Table 1). He was subsequently started on meningitis dose of Cefotaxime, in addition to Gentamycin ophthalmic drops while waiting for the previous cultures' results. Azithromycin was added as well to cover the possibility of an associated chlamydial infection.

The eye swab culture revealed Neisseria gonorrhea, which was sensitive to Cefotaxime, so the antibiotic was continued while waiting for the results of the CSF culture.

Blood and urine cultures were negative. The CSF culture revealed Gram-negative rods after one day, which was identified as *S. maltophilia* on day 5 of admission. The organism was sensitive to Trimethoprim-Sulfamethoxazole (TMP-SMX).

Once the diagnosis of *S. maltophilia meningitis* was identified, Cefotaxime was stopped and the baby was started on TMP-SMX and Ciprofloxacin. Since there are no clear guidelines on how to treat *S. maltophilia* meningitis in neonates, we extrapolated our management plan from that of other Gram-negative meningitis. Therefore, CSF was repeated at 2 days of antibiotics to confirm sterility. Because *S. maltophilia* meningitis is very rare and there are no clear guidelines on the duration of therapy, the treating team decided to repeat CSF studies one more time toward the end of the third week of antibiotics. The last CSF studies were completely normal, and the culture was negative.

The baby's head circumference was measured daily during the hospital stay and remained normal. Cranial ultrasound scan was normal.

The little boy recovered from the infection uneventfully and a follow-up visit of the baby 1 week after discharge was reassuring. His parents received treatment for gonorrhea and they were screened for other sexually transmitted diseases. 

## 3. Discussion

Populations at risk for *S. maltophilia* meningitis are typically immunocompromised patients, those who had neurosurgical procedures, preterm babies, and patients in need of prolonged hospitalization [2]. To our knowledge, only seven reports of *S. maltophilia* meningitis have been published to date, all of who had at least one of the above risk factors (Table 2). Our patient seems to be unique since he had none of the previous risk factors.

*S. maltophilia* is well known to be resistant to several antibiotics that are commonly used empirically for nosocomial infections. Mechanisms of resistance include production of beta-lactamase, efflux, biofilm formation, and aminoglycoside-modifying enzyme activity [7]. Another alarming feature of this pathogen is the significant heterogeneity among its isolates with high rate of genetic mutation [10].

The treatment of choice for *S. maltophilia* is Trimethoprim-Sulfamethoxazole (TMP-TMX), based on in vitro susceptibility tests and good clinical response reported in the past. Ciprofloxacin, Ceftazedime, and Ticarcillin/Clavulanate as monotherapy or in combination with other agents have been used with success. The optimal duration of therapy for *S. maltophilia* meningitis has not been well studied. We believe it should be similar to the duration used when treating other Gram-negative meningitis (i.e., at least three weeks). Likewise, we recommend obtaining CSF studies 48 hours after starting antibiotics to confirm sterility, and towards the end of the therapy to confirm normalization of all the CSF indices.

## 4. Conclusion

*Stenotrophomonas maltophilia* has received rising attention in the recent years since it is known as an evolving multidrug resistant organism. It is commonly identified as a cause of nosocomial infections; however, community-acquired infections are increasingly being reported. Although *S. maltophilia* meningitis continues to be rare in pediatric population, clinicians should be aware of it as a possible causative organism of meningitis, even in the absence of its known risk factors. We believe there is a lot yet to be learned about *S. maltophilia* and its associated clinical spectrum and appropriate duration of therapy for each condition.

## Figures and Tables

**Table 1:** Laboratory investigations performed at the time of admission.

Test	Results
Blood	
WBC	17.1 × 10 ^ 9/L
PMNs (%)	6.15 × 10 ^ 9/L (36%)
Lymphocytes (%)	8.03 × 10 ^ 9/L (47%), atypical 4%
Monocytes (%)	1.54 × 10 ^ 9/L (9%)
Hb (Hct)	177 g/L (0.50)
Platelets	392 x 10 ^ 9/L

Urine	
WBCs	<5
RBCs	<1
Nitrite/Leuk. Est.	-/-

CSF	
WBC	14 cells/mm^3^ (65% lymphocytes, 35% monocytes)
RBCs	101 cells/mm^3^
Color, clarity	Colorless, clear

WBC: white blood counts, PMNs: polymorph nuclear cells, Hb: hemoglobin, Hct: hematocrit, Leuk. Est.: leukocyte esterase, and CSF: cerebrospinal fluid.

**Table 2:** Reported pediatric cases of *S. maltophilia* meningitis.

Case	Age, gender	Risk factor	Origin	Treatment	Outcome
Denis et al. [3]	8 mo, M 13 mo, F	None None	Community	Ampicillin + Colistin Chloramphenicol + Sulphadoxine	Died Recovered
Sarvamangala Devi et al. [4]	7 days, M	Premature	Community	None	Died
Wen-Tsung et al. [5]	4 days, F	Premature	Nosocomial	Ciprofloxacin	Recovered
Rojas et al. [6]	12 days, M	Premature, EVD, ICH	Nosocomial	TMP-SMX and Ciprofloxacin	Recovered
Sood et al. [7]	6 months, M	Premature, VP shunt insertion	Nosocomial	Amikacin and TMP-SMX	Recovered
Correia et al. [8]	4 years, M	Premature, VP shunt, EVD	Nosocomial	TMP-SMX, Ceftazidime, and Levofloxacin	Recovered
Tandel et al. [9]	5 months, M	EVD	Nosocomial	TMP-SMX	Recovered

VP: ventricular-peritoneal, EVD: external ventricular device, and ICH: intracerebral hemorrhage.
